# Detour ahead: possible causes of corticospinal tract truncation in upper motor neuron–predominant amyotrophic lateral sclerosis

**DOI:** 10.1093/braincomms/fcaf419

**Published:** 2025-10-27

**Authors:** Venkateswaran Rajagopalan, Erik P Pioro

**Affiliations:** Department of Electrical and Electronics Engineering, Birla Institute of Technology and Science Pilani, Hyderabad Campus, Hyderabad 500078, India; Neuromuscular Center, Department of Neurology, Neurological Institute, Cleveland Clinic, Cleveland, OH 44195, USA; Department of Neurosciences, Lerner Research Institute, Cleveland Clinic, Cleveland, OH 44195, USA; Department of Medicine (Neurology), Djavad Mowafaghian Centre for Brain Health, University of British Columbia, Vancouver, BC, V6T 1Z3, Canada

**Keywords:** DTI, CST, ALS, phenotypes

## Abstract

Clinical diagnosis of amyotrophic lateral sclerosis (ALS) depends on finding evidence of combined of upper motor neuron (UMN) and lower motor neuron degeneration. T2- and proton density (PD)-weighted images reveal intracranial corticospinal tract (CST) hyperintensity in some UMN-predominant ALS patients who also exhibit faster disease progression compared to those without CST hyperintensity. Our previous study identified CST fibre tract truncation in a subset of UMN-predominant ALS patients, both with and without CST hyperintensity. In this study, we investigated the underlying cause(s) of CST fibre tract truncation in such ALS patients. Routine clinical diffusion tensor imaging (DTI) scans were acquired in 14 neurologic controls and 45 ALS patients using a 1.5 T magnetic resonance imaging (MRI) scanner. UMN-predominant ALS patients were categorized into two subgroups based on their clinically-acquired conventional MRI findings. DTI reconstruction was performed using both single-exponential and bi-exponential fitting approaches (the latter for free water [FW] estimation). DTI along the perivascular space (ALPS) index was also measured. CST fibres were reconstructed using tractography in both control and ALS subgroups. In CST-truncated ALS patients, the fibres deviated from their normal trajectory and entered the superior longitudinal fasciculus (SLF) at the level of the centrum semiovale, resulting in apparent truncation and overlap with the SLF. Axial diffusivity, radial diffusivity, FW content, and mean diffusivity values were normal along the expected CST pathway in cases of truncation, suggesting that axonal or myelin degeneration, inflammation, or oedema were unlikely to be responsible for CST truncation. ALPS index was significantly increased in CST-truncated patients compared to those without CST truncation. Based on these results, we hypothesize that impaired axonal guidance mechanisms or dysfunction of the glymphatic system may contribute to CST fibre tract truncation in ALS.

## Teaser

MRI shows corticospinal tract (CST) truncation in some amyotrophic lateral sclerosis patients with CST hyperintensity. Virtual CST fibers in such patients deviate from their expected brain trajectory by entering the superior longitudinal fasciculus. This disruption appears unrelated to axonal degeneration or inflammation/edema, but rather may reflect dysfunction in glymphatic drainage.

## Graphical Abstract

**Graphical Abstract:**
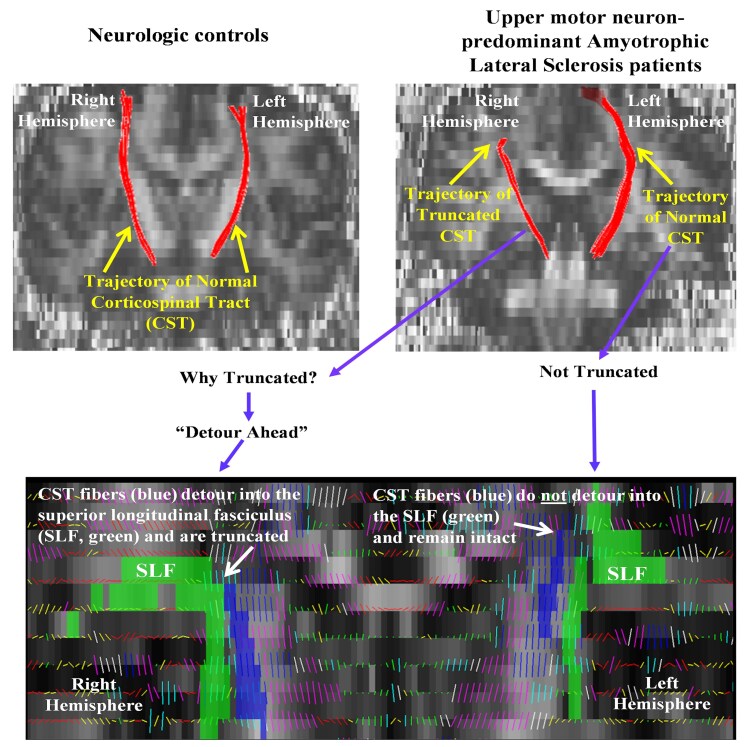

## Introduction

Amyotrophic lateral sclerosis (ALS) is a heterogeneously complex, incurable and fatal neurodegenerative disease. Diagnosis of ALS depends on the presence of specific progressive signs and symptoms, in the absence of mimicking conditions.^1^ Co-occurrence of upper motor neuron (UMN) and lower motor neuron (LMN) signs is essential for the diagnosis of ALS, in accordance with the revised El Escorial criteria.^2^ Degeneration of the corticospinal tract (CST)—which originates in the precentral gyrus (PrG) and passes through the posterior limb of the internal capsule (PLIC) to the spinal cord^3^ —is a pathological hallmark of the UMN degeneration in ALS. However, because no non-invasive objective test currently exists to detect UMN dysfunction,^1^ ALS diagnosis is often delayed.^4^ Therefore, identifying non-invasive neuroimaging biomarkers of UMN degeneration is essential to enable early diagnosis, distinguish between disease subtypes, monitor disease progression, and assess the effectiveness of therapeutic interventions.

A recent study reported that the proportion of ALS patients with CST hyperintensity on T2-weighted images was higher in those classified as having definite ALS compared to those with an indefinite diagnosis.^5^ Over several studies, approximatlely 40% (median value) of ALS patients with predominant UMN signs have been found to exhibit bilateral CST hyperintensity on conventional T2-, proton density (PD)-weighted, and fluid-attenuated inversion recovery (FLAIR) images.^1^ However, we also found a subgroup of UMN-predominant ALS patients with similar clinical features who lack CST hyperintensity (ALS-CST–). Those with CST hyperintensity (ALS-CST+) were significantly younger,^1,6^ exhibited faster disease progression^6^ and shorter survival^6^ than ALS-CST− patients. The reason for this observed difference among the ALS patients is unclear, and the underlying pathological substrate for these imaging differences remains unknown.

Previous studies have usually identified CST hyperintensity in ALS patients qualitatively—based on visual evaluation—using conventional MRI (cMRI) such as T2-weighted, PD-weighted, and FLAIR images.^1,3,5,7,8^ These approaches are subject to operator bias and do not yield quantitative information about the neuronal changes underlying the signal abnormalities. Diffusion tensor imaging (DTI), in contrast, was used to quantitatively evaluate central nervous system pathophysiological changes in ALS patients.^9,10^ A key advantage of DTI is its ability to reconstruct virtual neuronal fibre tracts through a technique known as ‘tractography’, which is not feasible with cMRI. In our previous study^11^ using diffusion tensor tractography (DTT), we found CST fibre tract absence or truncation between the centrum semiovale/top of lateral ventricle (CSoLV) level and the precentral gyrus in approximately 43% of ALS-CST+ patients and 17% of the ALS-CST− patients.

ALS patients with CST truncation were younger (*P* = 0.05), had significantly shorter disease duration (*P* = 0.001) and faster disease progression (*P* = 0.001) when compared to ALS patients without CST truncation. However, the reason for CST truncation was not addressed in our previous study. Therefore, the aim of the current investigation is to explore the underlying mechanisms of CST fibre tract truncation at the level of CSoLV in ALS patients. We believe that elucidating this mechanism may offer novel insights into the pathophysiological processes driving ALS in this patient population.

## Materials and methods

### Patient demographics and data acquisition

DTI data obtained as part of routine clinical neuroimaging evaluation were approved for storage and analysis as de-identified images by the Institutional Review Board at the Cleveland Clinic, following verbal patient consent. Informed consent was obtained in accordance with the Declaration of Helsinki (1991) from the patient/kin. DTI scans were acquired from 14 neurological controls and 45 ALS patients categorized into the following clinical phenotypes: UMN-predominant with CST hyperintensity (ALS-CST+) (*n* = 21, 14 male, 7 female; age 52.3 ± 11.4 years, mean ± standard deviation (SD), and UMN-predominant without CST hyperintensity (ALS-CST−) (*n* = 24, 13 male, 11 female; age 58.3 ± 11.4 years).

At the time of MRI, UMN-predominant ALS patients had prominent spasticity, hyperreflexia, and pathologic reflexes but no or only restricted LMN signs detected clinically or electrodiagnostically at a single neuraxial (bulbar, cervical, thoracic or lumbosacral) level. On clinical follow-up, however, LMN signs evolved (on a background of UMN signs) at two or more neuraxial levels to meet the diagnostic revised El Escorial criteria^2^ of at least clinically probable ALS in all ALS-CST+ and ALS-CST− patients. An El Escorial score with higher values representing more widespread UMN and LMN abnormalities across the four neuraxial levels was calculated from the El Escorial scale, as described previously.^12^ Loss of motor function at each neuraxial level is reflected by reduced score of the revised ALS functional rating (ALSFRS-R) scale.^13^ Therefore, monthly disease progression rate, as described elsewhere,^14^ was calculated by dividing the total number of lost ALSFRS-R points from normal (48) by the number of months from symptom onset, as shown below:


$$\begin{array}{r} \;\;\mathrm{Disease}\;\mathrm{Progression}\;\mathrm{Rate}\;=\;\frac{\left(48\;\text{–}\;\mathrm{ALSFRS}\text{-}R\;\mathrm{at}\;\mathrm{MRI}\right)}{\mathrm{Duration}\;\mathrm{of}\;\mathrm{symptoms}\;(\mathrm{months})} \end{array}$$

CST hyperintensity was defined as an easily visible increased signal along the intracranial CST especially in the PLIC and cerebral peduncle in both T2- and PD-weighted images. Clinical measures for ALS patients are provided in Table 1.

**Table 1:** Clinical parameters of ALS patients at time of MRI

Clinical measure/ALS subgroups	ALS-CST+ Mean ± SD	ALS-CST− Mean ± SD	Significance (*P* value)
El Escorial criteria score	1.81 ± 0.98	1.37 ± 0.82	NS
Duration of symptoms prior to MRI (months)	9.6 ± 5.5	36.4 ± 44.2	< 0.001
ALSFRS-R score	34.6 ± 7.8	34.1 ± 8.1	NS
Disease progression rate (monthly)	1.38 ± 1.64	0.46 ± 0.43	0.001

SD—Standard Deviation.

NS—Not Significant.

ALS-CST+—ALS patients with predominant upper motor neuron (UMN) signs and hyperintense signal along the corticospinal tract (CST) (on conventional proton density (PD) and T2-weighted images) and no clinical dementia.

ALS-CST−—ALS patients with predominant UMN signs without CST hyperintensity and no clinical dementia.

ALSFRS-R—Revised ALS functional rating scale.

### Imaging protocol

DTI data were acquired on a 1.5T magnet (Siemens Symphony, Erlangen, Germany) using a routine clinical imaging protocol. A single-shot echo planar imaging (SS-EPI) sequence was employed along 12 diffusion-weighted directions (b = 1000 s/mm^2^) and one b = 0 s/mm^2^. Imaging parameters were: 30 slices, 4-mm slice thickness, 1.9 × 1.9 mm in-plane resolution; repetition time TR = 6000 ms, echo time TE = 121 ms, EPI factor = 128, and total scan time = 7.54 min. Gradient-echo field maps were acquired to correct for susceptibility-induced geometrical distortions. Field map parameters were: 30 slices, 4 mm slice thickness, 4 mm slice gap, TR = 500 ms, TEs = 6.11 ms and 10.87 ms. CST hyperintensity was evaluated on T2- and PD-weighted images acquired via a dual-echo fast spin echo sequence, with parameters: 40 contiguous slices, 4 mm slice thickness, 0.9 × 0.9 mm in-plane resolution; TR = 3900 ms, TEs = 26 and 104 ms, total scan time = 3.5 min.

### Data processing

#### Single compartment DTI model

Diffusion weighted images (DWI) were first corrected for susceptibility artefacts using FSL’s (https://fsl.fmrib.ox.ac.uk/fsl/docs/index.html) FUGUE^15-17^ and then for eddy current-induced distortions. The b-matrix was rotated to preserve correct orientation.^18,19^ DTI Studio^20^ (https://www.mristudio.org/installation.html) was used to process the corrected images. Diffusion tensors for each voxel were computed using a multivariate linear least-squares fit and diagonalized to obtain principal eigenvalues and eigenvectors. Maps for diffusion metrics—fractional anisotropy (FA), mean diffusivity (MD), axial diffusivity (AD) and radial diffusivity (RD)—were obtained.

#### Free water eliminated two-compartment DTI model

Given our single shell DWI acquisition protocol, commonly used in routine clinical imaging, a two-compartment model was fitted using a regularized gradient descent algorithm.^21^ The model includes a tissue diffusion tensor (D_t_) and a free water (FW) diffusion tensor (D_w_), using the equation:


$$A=f\times exp(-b_{j}x_{j}^{T}D_{t}x_{j})+(1-f)\times exp(-b_{j}D_{w})$$

Here, A = S_j_/S_0_, where S_j_ is the signal measured with diffusion gradient j (direction x_j,_ b-value b_j_), S_0_ is the signal without diffusion weighting (b = 0), f is the effective tissue-water fraction, and (1−f) is the FW fraction. Pre-processed DWI, a binary brain mask, and the corrected b-matrix were input into the FW estimation code obtained from https://github.com/mvgolub/FW-DTI-Beltrami using DIPY libraries^21^ (https://dipy.org/). After tensor fitting, maps of FA, free water-eliminated (FWE) FA, AD, RD, MD and FW were generated for the whole brain. The FW map reflects the fraction of unconfined water molecules in each voxel.

#### Diffusion tensor tractography

Virtual neuronal fibres were reconstructed using DTI Studio’s fibre assignment by continuous tracking (FACT) algorithm.^20,22^ Fibre tracking was initiated from every voxel^22^ with FA ≥ 0, with a termination threshold of FA = 0.2 and maximum turning angle of 41^°^. The CST and superior longitudinal fasciculus (SLF) were reconstructed bilaterally using region-of-interest (ROI) placement as described in Wakana *et al*.^22^ Tractography was performed using both standard FA and FWE-FA images.

#### Measuring diffusion tensor metrics at the CST truncation level

**Figure 1:**
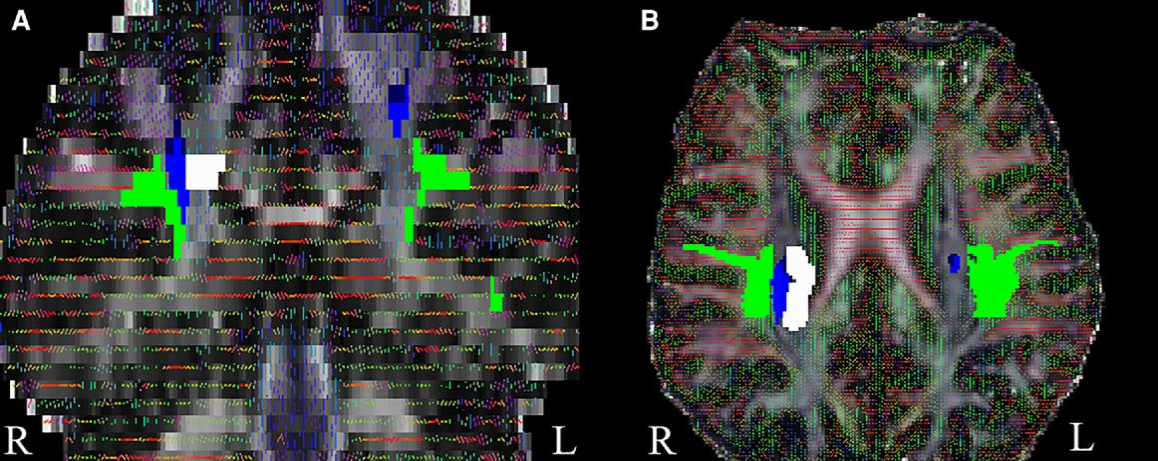
**Course deviation of the CST in ALS**. Coronal (**A**) and axial (**B**) MRI views show deviated location of tractography-identified right corticospinal tract (CST, blue) adjacent to the superior longitudinal fasciculus (green). This is in contrast to the expected (normal) location of the CST (white) averaged from several neurologic controls.

CST fibre tracts from all 14 neurological controls (both hemispheres) were registered to MNI space using FSL, and an average CST map was generated. These average tracts were then warped to each ALS patient’s native space and thresholded at a probability > 0.5 to isolate core CST regions and remove registration artefacts. These average tracts were superimposed on each ALS patient’s CST and SLF reconstructions. In ALS patients with CST truncation, these control-based tracts identified where the CST was expected but instead deviated towards the SLF. Guided by these deviations, ROIs were placed adjacent to the diverted CSTs to measure FA, FWE-FA, AD, RD, MD and FW values (see Fig. 1 for a representative example). These metrics were used to understand whether the CST diversion resulted from inflammation (FW and MD), axonal/myelin damage (AD and RD) or white matter (WM) integrity compromise (FA and FWE FA).

**Figure 2:**
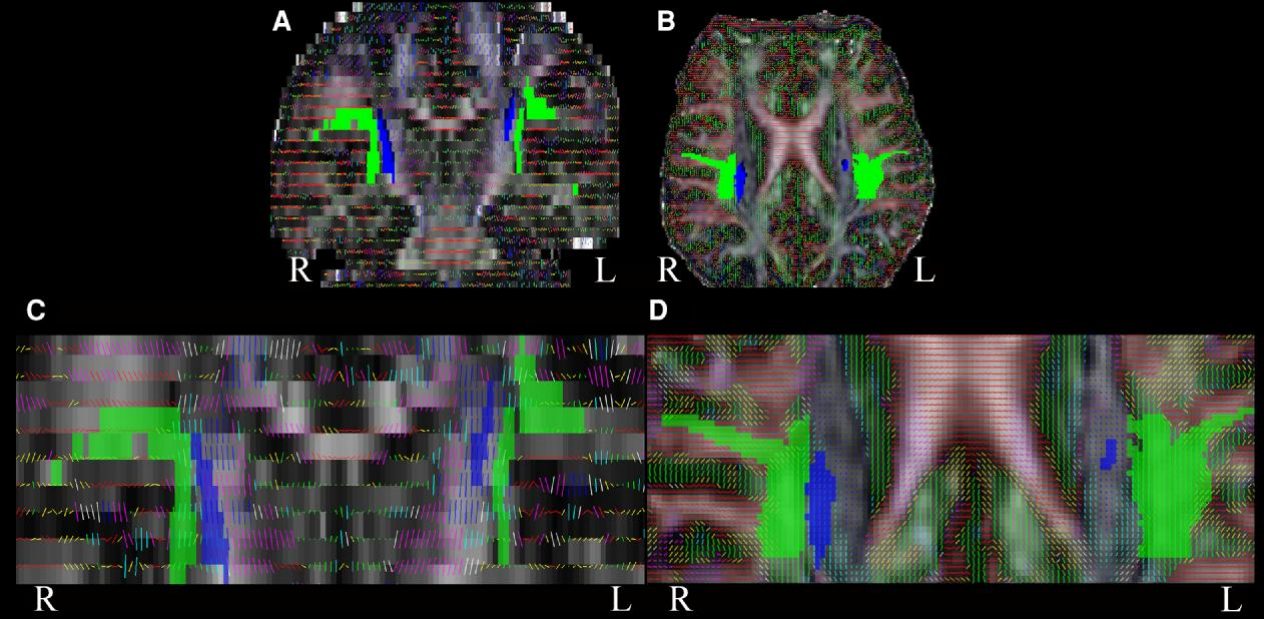
**Eigenvectors overlying deviated CST in ALS**. Coronal (**A**) and axial (**B**) views of a brain fractional anisotropy MRI from an ALS patient with corticospinal tract (CST) hyperintensity (ALS-CST+), show tractography-derived CST (blue) and superior longitudinal fasciculus (SLF, green) at the level of centrum semiovale at top of lateral ventricle. Higher magnification coronal (**C**) and axial (**D**) views, where clearly seen overlying eigenvectors show virtual CST fibres in the right hemisphere entering the SLF and becoming truncated, while those in the left hemisphere do not.

AD, RD, and MD values measured in each voxel of ROIs placed adjacent to the CST in the slices showing truncation in ALS-CST+ (9 patients) and ALS-CST− (4 patients) were manually compared (visually verified) with corresponding values, i.e. whether they fell within the mean ± standard deviation values of neurologic controls at the CSoLV level reported in our previous study^12^ (see Figs 1–4 in that publication). Similarly, FW values in each voxel of the ROIs were manually compared (visually verified) for any abnormality, defined as FW values > 0.3 (as seen in oedema) and in cerebrospinal fluid (∼0.9). In these ROIs placed adjacent to the CST in the slices showing truncation, FW values were found to be within the normal range of 0.02 to 0.21.

#### Diffusion along perivascular space data processing

The ALPS index was computed in ALS-CST+ and ALS-CST− subgroups using the automated alps.sh script available from GitHub (https://github.com/gbarisano/alps/).^23^ Briefly, pre-processing included denoising and Gibbs unringing, and eddy current correction. DTI model fitting was conducted using FSL’s dtifit to generate dxx, dyy, and dzz tensor maps, which were used to calculate ALPS index. Images were registered to JHU template space using FSL, and ROIs were automatically placed to calculate left and right hemisphere ALPS values. Depending normality assumptions, either the Mann–Whitney U test or Student’s *t*-test was used to compare mean ALPS values between the CST-truncated patients (across both ALS-CST+ and ALS-CST− groups) and non-truncated patients (from both groups as well).

#### Clinical measures

Disease duration, ALSFRS-R score, and monthly disease progression rate were compared between ALS subgroups, as well as between patients with and without CST truncation, using either the Mann–Whitney U test or Student’s *t*-test, depending on whether the data met assumptions of normal distribution.

## Results

### Tractography

While reconstructing virtual CST fibres in the WM subcortical to the primary motor cortex (subPMC) using tractography based on FA maps not corrected for FW, we observed absence or ‘truncation’ of CST fibres between the CSoLV level and primary motor cortex (PMC) in some patients from both UMN-predominant ALS subgroups. Truncation was noted primarily in the ALS-CST+ subgroup (9 of 21 patients, 42.8%) and significantly less frequently (*P* < 0.014) in the ALS-CST− subgroup (4 of 24 patients, 16.6%). Notably, CST was not truncated in any of the neurologic controls. As demonstrated in our previous study,^11^ virtual CST fibres connecting the PMC to subcortical WM were preserved in neurologic controls but disrupted in ALS patients due to truncation.

In CST truncated patients, FA values in some voxels within ROIs adjacent to the CST on truncated slices (Fig. 1) were found to be below the tractography threshold of 0.2. To investigate whether these low FA values caused the observed truncation, we compared them with the corresponding FWE FA values in the same voxels. The FWE FA values exceeded 0.2, suggesting that FW contamination may have led to underestimation of FA. Consequently, we attempted CST reconstruction using the FWE FA maps, expecting that tracts might now extend without interruption. However, in all but one ALS-CST+ patient, CST fibre tracts remained truncated above the CSoLV level, persisting in 8 of 9 ALS-CST+ patients and all 4 of the ALS-CST− patients with initial truncation.

AD, RD, and MD values were measured in ROIs adjacent to the CST in the slices showing truncation in ALS-CST+ (9 patients) and ALS-CST− (4 patients), and were compared to corresponding values from neurologic controls at the CSoLV level based on our previous study^12^ (see Figs 1–4 in that publication). None of the ALS-CST+ or ALS-CST− patients exhibited abnormal AD, RD or MD values in these ROIs, suggesting that CST truncation was not due to axonal or myelin damage. FW values in these ROIs ranged from 0.02 to 0.21, markedly lower than values typical of cerebrospinal fluid (∼0.9) or oedema (>0.3),^24^ indicating that inflammation or oedema was also unlikely to be the cause.

Visual inspection of MRI slices where CST truncation occurred revealed that CST fibres deviated from their normal trajectory towards the subPMC and instead entered voxels with principal eigenvectors aligned in the anterior–posterior direction (visualized as green in colour-coded FA maps; Fig. 2). Because the SLF is also present at the CSoLV level, we reconstructed SLF fibres to examine whether they intersected or interfered with the CST path. In all of the 13 CST-truncated patients (9 ALS-CST+ and 4 ALS-CST− patients) the CST tracts entered (deviated from their original path towards subPMC) the voxels close to SLF, while in 2 of these 13 patients, the CST tracts overlapped with the SLF, appearing to terminate within it—thereby resulting in truncation.

No statistically significant differences in the ALPS index between CST-truncated patients (left hemisphere: 1.23 ± 0.13; right hemisphere 1.22 ± 0.11; mean ± SD) and CST non-truncated patients (left: 1.20 ± 0.17; right: 1.20 ± 0.17) were noted. Similarly, among ALS-CST+ patients, the ALPS index was 1.26 ± 0.12 (left) and 1.25 ± 0.10 (right) in truncated cases and 1.22 ± 0.15 (left) and 1.22 ± 0.15 (right) in non-truncated cases. For ALS-CST− patients, the index was 1.14 ± 0.10 (left) and 1.14 ± 0.08 (right) in truncated cases and 1.19 ± 0.18 (left) and 1.20 ± 0.18 (right) in non-truncated cases.

DTT revealed significantly more frequent CST truncation (*P* <0.014) in ALS-CST+ compared to ALS-CST− patients. When compared with ALS patients without CST truncation, those with truncation were younger (*P* = 0.05), had significantly shorter disease duration (*P* = 0.001) and exhibited a faster rate of disease progression (*P* =0.001).

### Clinical measures

Symptom duration prior to MRI was significantly shorter (*P* < 0.001) in the ALS-CST+ subgroup compared to the ALS-CST− subgroup. Notably, ALS-CST+ patients had significantly faster disease progression rates (*P* = 0.001) at the time of MRI than ALS-CST− patients, and shorter survival times, as reported previously.^6^ Additionally, ALS-CST+ patients tended to be younger than their ALS-CST− counterparts (*P* = 0.05).

## Discussion

This study demonstrates that virtual subcortical WM fibres appear disrupted (‘truncated’) in the following ways: (1) truncation occurs in the descending CST of ALS patients compared to neurologic controls; (2) this disruption is due to CST fibres deviating from their expected trajectory between the CSoLV and subPMC; (3) CST fibres enter the region of the SLF; and (4) this deviation and resulting truncation do not appear to be caused by FW contamination, axonal or myelin degeneration, or inflammation/oedema as the values for AD, RD, MD and FW are all within the normal ranges in the affected CST regions on the disrupted MRI slices.

DTT detects virtual CST fibre disruption in UMN predominant ALS patients, whereas CST truncation is not observed in any of the neurologic controls (*n* = 14). FWE-FA maps also failed to reconstruct CSTs above the CSoLV level in patients with truncation, suggesting that if the original FA map was affected by oedema/inflammation, correction for FW would have resolved the artefact. According to Parker *et al*.,^24^ FW values greater than 0.3 are indicative of oedema. In our study, FW values in and around the CST-truncated regions were below 0.23, supporting the absence of oedema or inflammation that could explain CST deviation into the SLF region.

Additionally, the presence of normal AD and RD values near the truncated CST segments suggests that axonal or myelin injury did not account for the tract deviation. Normal MD values further support the absence of WM damage or associated pathological processes such as inflammation or gliosis. We also evaluated the possibility that CST truncation resulted from technical limitations, including poor DTI data quality (e.g. low signal-to-noise ratio, anisotropic voxels, limited resolution) or from tractography reconstruction algorithms. However, since neurologic controls did not display CST truncation, these technical factors can be ruled out. Budde *et al*.^25^ reported that gliosis can increase diffusion anisotropy due to reactive astrocytes and that glial scarring can lead to spurious tracts in tractography. However, we did not observe any spurious fibre tracts in the CST-truncated slices, suggesting that gliosis did not contribute to the observed truncation in our cohort.

We also investigated the role of glymphatic system dysfunction using the ALPS index and found it to be higher in the CST-truncated group compared to the non-truncated ALS patients. When stratified by subgroup, ALS-CST+ patients with CST truncation exhibited a higher ALPS index than their non-truncated counterparts, while the reverse was observed in ALS-CST− patients. Although this difference was not statistically significant when combining both subgroups, the elevated ALPS index in CST-truncated patients suggests potential disruption in glymphatic function at the subcortical periventricular level.

Based on these findings, we speculate that the ALS disease process may differentially affect glymphatic function and axonal growth factors in CST-truncated patients compared to those without truncation, causing the CST tracts to diverge into the SLF tract region. Future studies integrating histochemical markers with DTI are needed to validate this hypothesis. These findings suggest a potential microanatomic pathology extrinsic to the CST itself, which disrupts virtual fibre reconstruction.

Our previous study^11^ showed that CST truncation occurred in subcortical fibres projecting from the PMC but not those projecting to/from the primary sensory cortex, supporting the specificity of this phenomenon to motor pathways—consistent with the known relative sparing of sensory systems in ALS. Furthermore, we previously confirmed that the deterministic tractography FACT algorithm used was not responsible for the observed CST truncation, as similar findings were replicated using FSL's probabilistic tractography method. These results collectively indicate that neither the image acquisition protocol nor the processing algorithms accounted for CST truncation.

In summary, CST fibres deviated from their normal path towards the subPMC and entered the SLF tract region in a subset of ALS patients, with some tracts even overlapping with the SLF. This deviation was not due to artefacts in FA values, tractography methods, or microstructural changes such as axonal or myelin loss or inflammation/oedema. An elevated ALPS index in CST-truncated patients supports a role for impaired glymphatic function. The persistence of virtual CST truncation exclusively in ALS patients suggests a real microanatomic difference at the rostral extent of these fibres. However, future studies with larger imaging datasets will be necessary to validate the glymphatic dysfunction hypothesis and to correlate DTT findings with corresponding histopathological changes in post mortem tissue. Such studies will help elucidate this intriguing imaging phenomenon and strengthen its potential role in clinical diagnosis of rapidly progressing UMN-predominant ALS.

## Conclusion

In patients with CST truncation, the disruption appears to result from CST fibres deviating from their expected course and entering the SLF tract region. No evidence of axonal or myelin degeneration, nor inflammation or oedema, was observed along the original CST path to account for this deviation. An elevated ALPS index in CST-truncated patients—but not in those without truncation—suggests that the glymphatic system may be impaired in these individuals. We hypothesize that ALS-related disruptions in axonal growth factors and glymphatic clearance contribute to CST fibre misrouting. Further research integrating imaging with histopathology is needed to confirm these findings.

## Data Availability

Although access to the imaging datasets analysed in the current study can be requested, their proprietary nature will likely preclude sharing by the Cleveland Clinic.
